# Enhancing precision in vascular embolization: evaluating the effectiveness of the intentional early detachment technique with detachable coils in complex cases

**DOI:** 10.1186/s42155-024-00453-7

**Published:** 2024-04-25

**Authors:** Shojiro Oka, Shigeshi Kohno, Shigeki Arizono, Yasuyuki Onishi, Masaya Fumimoto, Atsushi Yoshida, Reiichi Ishikura, Kumiko Ando

**Affiliations:** 1 Department of Diagnostic Radiology, Kobe City Medical Center General Hospital, 2-1-1 Minatojimaminamimachi, Chuo-ku, Kobe, Hyogo 650-0047 Japan; 2 Department of Diagnostic Imaging and Nuclear Medicine, Kyoto University Graduate School of Medicine, 54 Kawahara-cho, Shogoin, Sakyo-ku, Kyoto, 606-8507 Japan

**Keywords:** Detachable coils, Vascular embolization, Tortuous pathways, Coil detachment, Saline flushing

## Abstract

**Background:**

This study aimed to assess the effectiveness and versatility of an intentional early detachment technique with detachable coils in addressing challenging vascular embolization scenarios. This novel approach aims to provide an alternative method for achieving precise coil placement when standard methods of detachable coil placement are ineffective owing to vascular anatomy or limited available equipment.

**Materials and methods:**

This retrospective study included 11 patients (nine males and two females; median age, 77 years) who underwent embolization procedures between October 2021 and December 2023 using the intentional early detachment technique through 1.6-Fr or 1.3-Fr microcatheters. In this technique, detachable coils were intentionally detached within the microcatheter and placed through saline flushing. The technique’s technical success, complications, and clinical success were evaluated.

**Results:**

The technique was applied in three distinct scenarios: tortuous vascular anatomy (four cases), inadequate system backup (three cases), and 1.3-Fr microcatheter use (four cases). The technical and clinical success rates were 100%. No complications were observed, and no cases of coil migration or malpositioning.

**Conclusion:**

The intentional early detachment technique is valuable for interventional radiologists and offers a solution for challenging vascular embolization scenarios. Its application is limited to specific circumstances; however, it can significantly enhance coil placement in complex cases, thereby contributing to improved patient care.

**Graphical Abstract:**

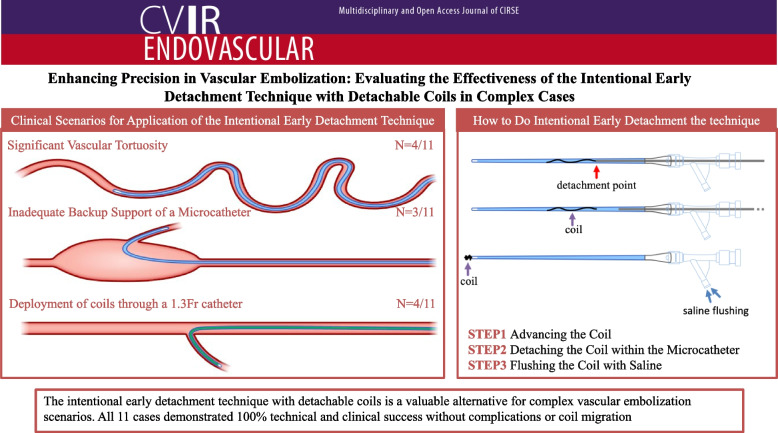

## Background

Since their development in 1991, detachable coils have been recognized for their efficacy and safety in cases of vascular embolization, especially in neurointervention [1–4]. These coils can be deployed with control and precision when attached to a delivery wire. They have become a crucial part of the intervention toolkit in cases where precision is essential, and downstream non-target embolization would be catastrophic [5]. However, despite their proven efficacy, detachable coils have limitations. There can be compatibility issues between microcoils and microcatheters, making it difficult to insert and deploy microcoils successfully in specific clinical situations, even when the possible combinations are configured by manufacturers [6]. Notably, the successful deployment of detachable coils can become challenging when dealing with tortuous vessels or when the backup support of the microcatheter is insufficient. In such situations, the precise placement of coils may be compromised, leading to undesired outcomes.

Recent advancements in catheter systems have facilitated the catheterization of tortuous or narrow branches using exceptionally thin microcatheters [7, 8]. Microcoils are compatible with most microcatheters if their primary diameters are smaller than the inner diameters of the microcatheter tips [6]. However, the narrow inner lumen of these thin catheters limits the types of coils that can be deployed. Notably, the use of coils is almost incompatible with 1.3-Fr microcatheters. Therefore, we devised an intentional early detachment technique with detachable coils to address these challenges. This novel technique offers a solution for the vascular embolization of tortuous or narrow branches only accessible with thin microcatheters. Interventional radiologists can achieve precise coil placement by intentionally detaching the detachable coils within the microcatheters and flushing them with saline. We explored the application of this intentional early detachment technique in 11 patients, each presenting unique challenges. Furthermore, our investigation aims to elucidate the effectiveness and versatility of this technique, providing interventional radiologists with a viable alternative for complex scenarios and ultimately enhancing patient care.

## Methods

### Patients

All procedures involving human participants followed the ethical standards of the institutional and/or national research committee and the 1964 Helsinki Declaration and its later amendments or comparable ethical standards. Our local Institutional Review Board approved the study (approval number: Zn240208) and waived the need for written informed consent. The intentional early detachment of detachable coils is an off-label technique, and this fact was explained to our institutional ethics committee. Between October 2021 and December 2023, 11 embolizations were performed on 11 patients (nine males and two females, with a median age of 77 years [range, 52–87]) using the intentional early detachment technique with detachable coils through a 1.6-Fr or 1.3-Fr microcatheter.

### Procedure

**Fig. 1:**
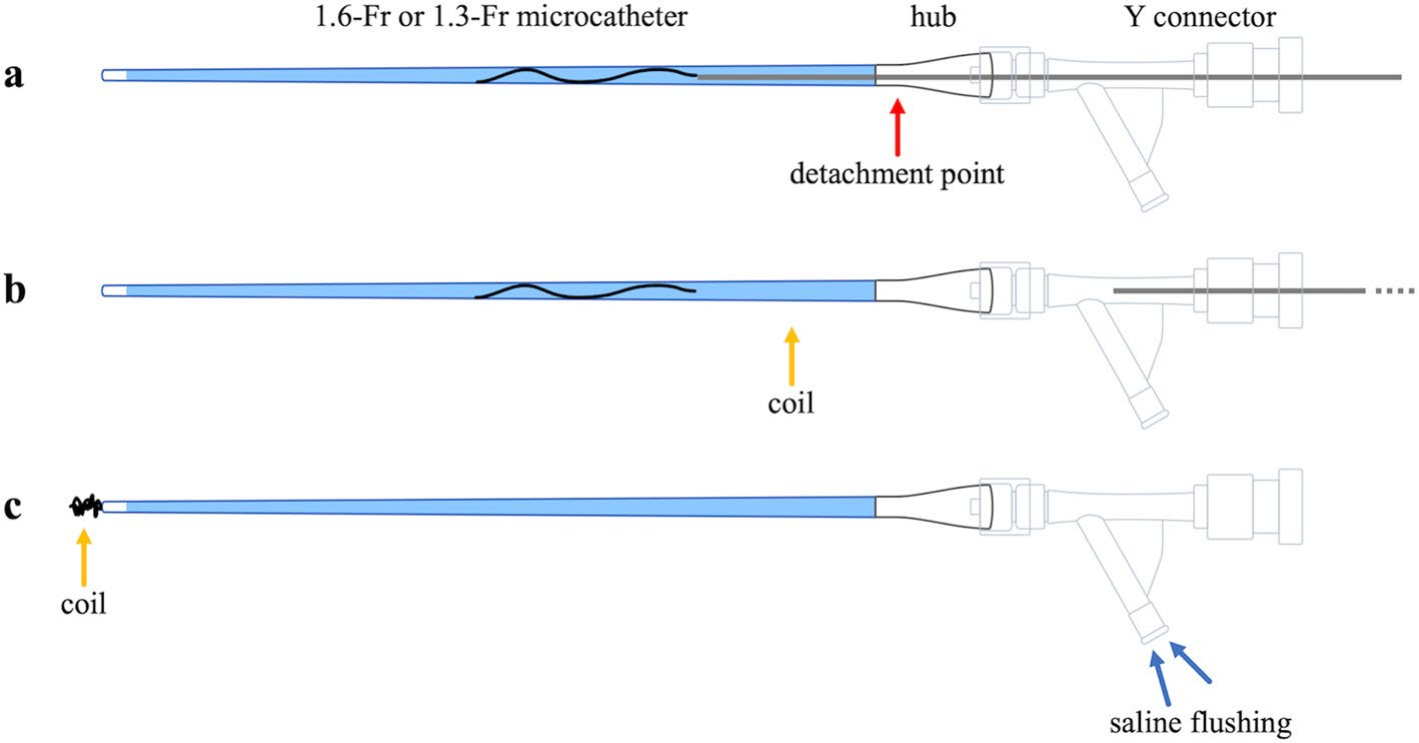
The intentional early detachment technique process. **a** advancing the detachable coil through the microcatheter until the delivery wire enters without resistance or the microcatheter’s kickback; **b** intentionally detaching the coil within the microcatheter; **c** completing the procedure with saline flushing to secure coil placement

**Fig. 2:**
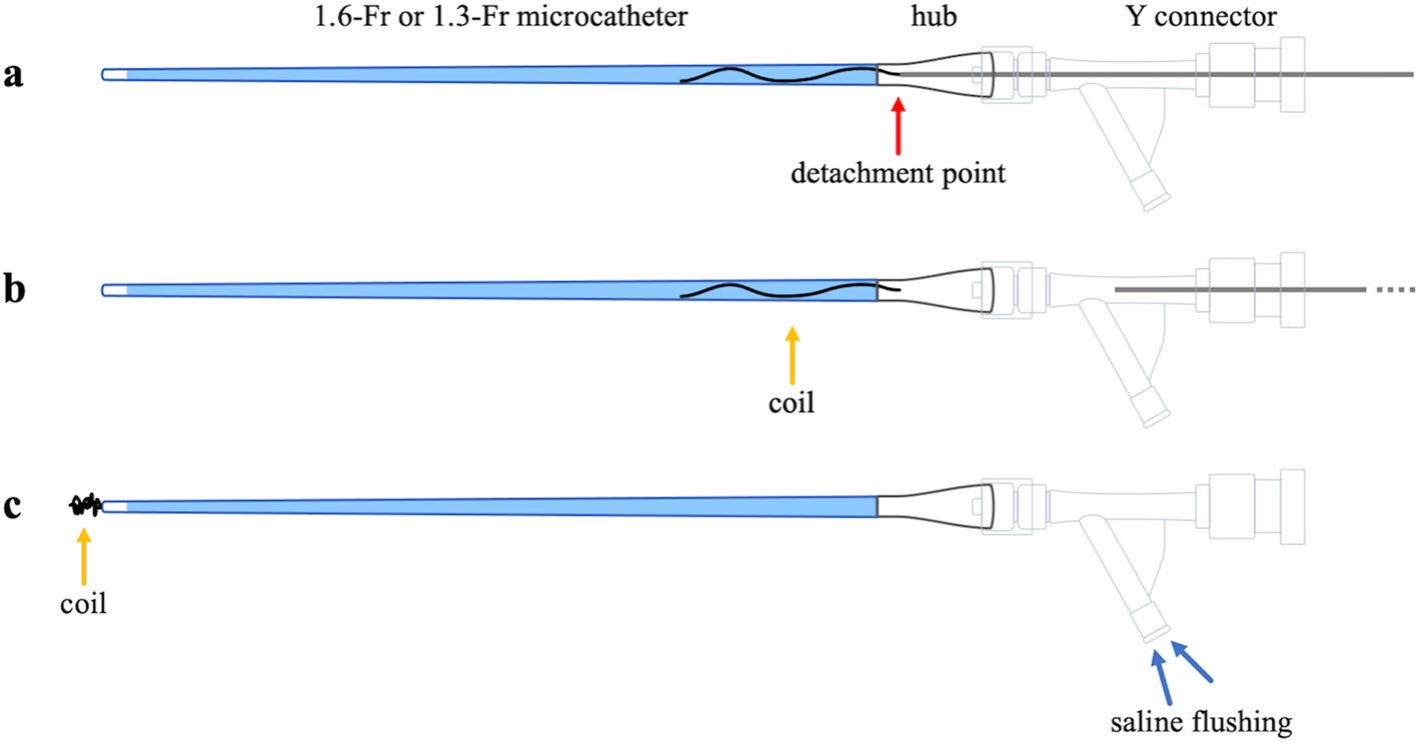
Using coils that cannot be detached within the catheter. **a** advancing only the coil portion of the detachable coil into the microcatheter, ensuring the detachment point (indicated by the red arrow) is just inside the catheter hub; **b** detachment is achieved at the designated point; **c** completing the procedure with saline flushing to secure coil placement

In all cases, this technique was used when embolizing the vessel using standard methods of detachable coil placement was impossible, and the operator considered embolization with a liquid embolic material inappropriate. We advanced the coil through the microcatheter until the delivery wire could enter without resistance or the microcatheter’s kickback. We then intentionally detached the coil within the microcatheter and flushed the coil with saline (Fig. 1). Notably, some electrically detachable coils, such as the iED (KANEKA Medics, Osaka, Japan) and Target coils (Stryker, Fremont, CA, USA) may not be detachable within the catheter. When using this type of coil, advancing only the coil portion into the microcatheter and ensuring that the detachment point was slightly within the catheter hub allowed for successful detachment (Fig. 2). The materials used in each case are summarized in Table 1. In all cases, the triaxial system, advancing a small microcatheter from a large microcatheter, was introduced initially because the target arteries were small and/or tortuous on preprocedural computed tomography images. We used a large microcatheter (Swift NINJA; SB KAWASUMI, Tokyo, Japan in seven cases, Masters HF; Asahi Intecc, Aichi, Japan in three cases, and Guidepost; Tokai Medical Products in one case) and a small microcatheter (Carnelian MARVEL S; Tokai Medical Products, Aichi, Japan in seven cases, Carnelian MARVEL S 1.3; Tokai Medical Products, Aichi, Japan in three cases, and DeFrictor Nano; Medico's Hirata, Osaka, Japan in one case). The 1.6-Fr microcatheters were advanced along CHIKAI V, CHIKAI X10 (Asahi Intecc, Aichi, Japan), and Traxcess (Terumo, Tokyo, Japan) microguidewires, whereas the 1.3-Fr microcatheters were advanced along the CHIKAI X10 microguidewire. The microguidewires used were CHIKAI V, CHIKAI X10 (Asahi Intecc, Aichi, Japan), and Traxcess (Terumo, Tokyo, Japan). Notably, various types of detachable coils were employed, including Target XL soft, Target Nano, Target Tetra (Stryker), SMART COIL (Penumbra, Alameda, CA, USA), AVENIR, AVENIR PICO (Wallaby Medical, Shanghai, China), AZUR soft 3D (Terumo, Tokyo, Japan), and iED coil (KANEKA Medix). The coil sizes ranged from 1 to 6 mm in diameter and 2 cm to 20 cm in length. However, the number of coils used per case varied from 1 to 8.

**Table 1:** Eleven patients who underwent coil embolization using the intentional early detachment technique with detachable coils

Patient No /Age /Sex	Embolized artery	Etiology	Coil: diameter in mm/ length in cm	No of coils	Microcatheters/ microguidewire	Reasons for intentional early detachment	Technical/Clinical success	Complication
1/52/M	AIPDA	Hemorrhage due to SAM	Avenir PICO: 1/2(× 2)	2	Carnelian MARVEL S 1.3, Swift Ninja/CHIKAI X10	Deployment of coils through a 1.3-Fr catheter	YES/YES	NO
2/69/M	3rd ICA	Preemptive embolization to prevent T2EL	Target XL: 5/10(× 2), 4/15(× 5) Azur soft3D: 4/10	8	Carnelian MARVEL S, Swift Ninja/Traxcess	Significant tortuosity in vascular anatomy	YES/YES	NO
3/74/M	Cystic artery	Idiopathic gallbladder bleeding	Target nano: 1/2(× 2)	2	Guidepost, DeFrictor Nano, Carnelian MARVEL S/CHIKAI X10	Deployment of coils through a 1.3-Fr catheter	YES/YES	NO
4/75/M	PIPDA	Hemorrhage within a pancreatic tumor	Target XL soft: 6/20	1	Carnelian MARVEL S, Swift Ninja/CHIKAI V, CHIKAI X10	Significant tortuosity in vascular anatomy	YES/YES	NO
5/75/F	DPA	True aneurysm due to MALS	Target nano: 1/2, 1/3(× 3)	4	Carnelian MARVEL S, Swift Ninja/CHIKAI V, CHIKAI X10	Inadequate system backup	YES/YES	NO
6/76/M	2nd LA	T2EL	Avenir: 4/20, 3/10, 4/13(× 2), 5/17(× 2) Target tetra: 3.5/8(× 2)	8	Carnelian MARVEL S 1.3, Masters HF/CHIKAI X10	Deployment of coils through a 1.3-Fr catheter	YES/YES	NO
7/79/F	PIPDA	Post-EST hemorrhage	SMART Coil: 1/2	1	Carnelian MARVEL S 1.3, Masters HF/CHIKAI X10	Deployment of coils through a 1.3-Fr catheter	YES/YES	NO
8/83/M	Iliolumbar artery	Lt-IIAA Embolization to prevent T2EL	Avenir: 3/10, 2/8, 2/6	3	Carnelian MARVEL S, Swift Ninja/CHIKAI V, CHIKAI X10	Inadequate system backup	YES/YES	NO
9/86/M	VR	Diverticular hemorrhage	Avenir: 1.5/3(× 2),2/4, 2/3(× 2) iED coil: 1/3	6	Carnelian MARVEL S, Masters HF/CHIKAI V, CHIKAI X10	Inadequate system backup	YES/YES	NO
10/86/M	4th LA	T2EL	Avenir: 4/20	1	Carnelian MARVEL S, Swift Ninja/CHIKAI X10	Significant tortuosity in vascular anatomy	YES/YES	NO
11/87/M	4th LA	T2EL	AZUR soft3D: 3/8 Avenir: 4/20	2	Carnelian MARVEL S, Swift Ninja/CHIKAI X10	Significant tortuosity in vascular anatomy	YES/YES	NO

Target XL soft/Nano/Tetra (Stryker, Fremont, CA, USA), SMART COIL (Penumbra, Alameda, CA, USA), AVENIR/AVENIR PICO (Wallaby Medical, Shanghai, China), AZUR soft 3D (Terumo, Tokyo, Japan), iED coil (KANEKA Medix, Osaka, Japan)

Carnelian MARVEL S/S 1.3 (Tokai Medical Products, Aichi, Japan), Swift NINJA (SB KAWASUMI, Tokyo, Japan), Masters HF (Asahi Intecc, Aichi, Japan), DeFrictor Nano (Medico's Hirata, Osaka, Japan), Guidepost (Tokai Medical Products), CHIKAI V/X10 (Asahi Intecc, Aichi, Japan), Traxcess (Terumo, Tokyo, Japan)

*AIPDA* anterior inferior pancreaticoduodenal artery, *PIPDA* posterior inferior pancreaticoduodenal artery, *DPA* dorsal pancreatic artery, *LA* lumbar artery, *MALS* median arcuate ligament syndrome, *EST* endoscopic sphincterotomy, *ICA* intercostal artery, *IIAA* internal iliac artery aneurysm, *VR* vasa recta, *T2EL* type 2 endoleak, *SAM* segmental arterial mediolysis

### Evaluation

The primary outcome parameter was technical success, defined as when the coils were delivered and placed successfully into the target vessel without migration. Complications and clinical success rates were also evaluated. Clinical success was defined as the complete cessation of blood flow at the target vessel through the post-embolization angiogram. Complications were assessed using the Cardiovascular and Interventional Radiological Society of Europe classification system [9]. Two radiologists with > 8 years of experience in diagnostic and interventional radiology interpreted all the images. Any disagreements or discrepancies were resolved by consensus.

## Results

**Fig. 3:**
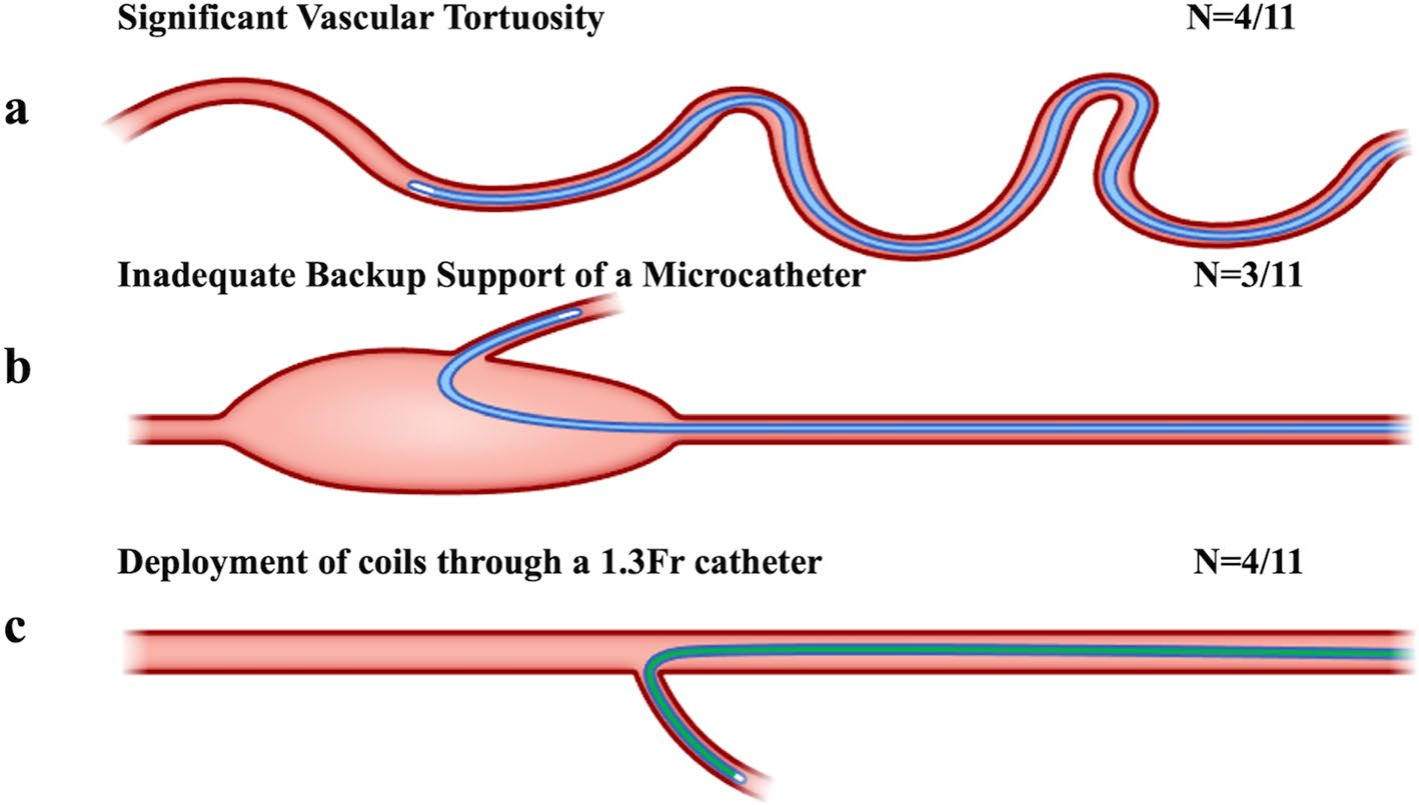
Clinical scenarios for applying the intentional early detachment technique. **a** cases in which the vascular anatomy exhibits significant tortuosity, challenging the process of pushing a detachable coil's delivery wire through microcatheters; **b** cases in which inadequate system backup results in microcatheter kickbacks during coil placement. Microcatheter kickback challenges the tight placement of detachable coils and poses a potential risk of catheter dislodgment into the mother vessel; **c** cases in which coils are deployed through a 1.3-Fr microcatheter

Notably, 11 patients (nine males and two females, with a median age of 77 years) underwent embolization procedures using the intentional early detachment technique. The patients' conditions and the vessels that underwent embolization are summarized in Table 1. The intentional early detachment technique was employed in three distinct clinical scenarios (Fig. 3): tortuous vascular anatomy (*n* = 4), inadequate system backup (*n* = 3), and using a 1.3-Fr microcatheter (*n* = 4).

**Fig. 4:**
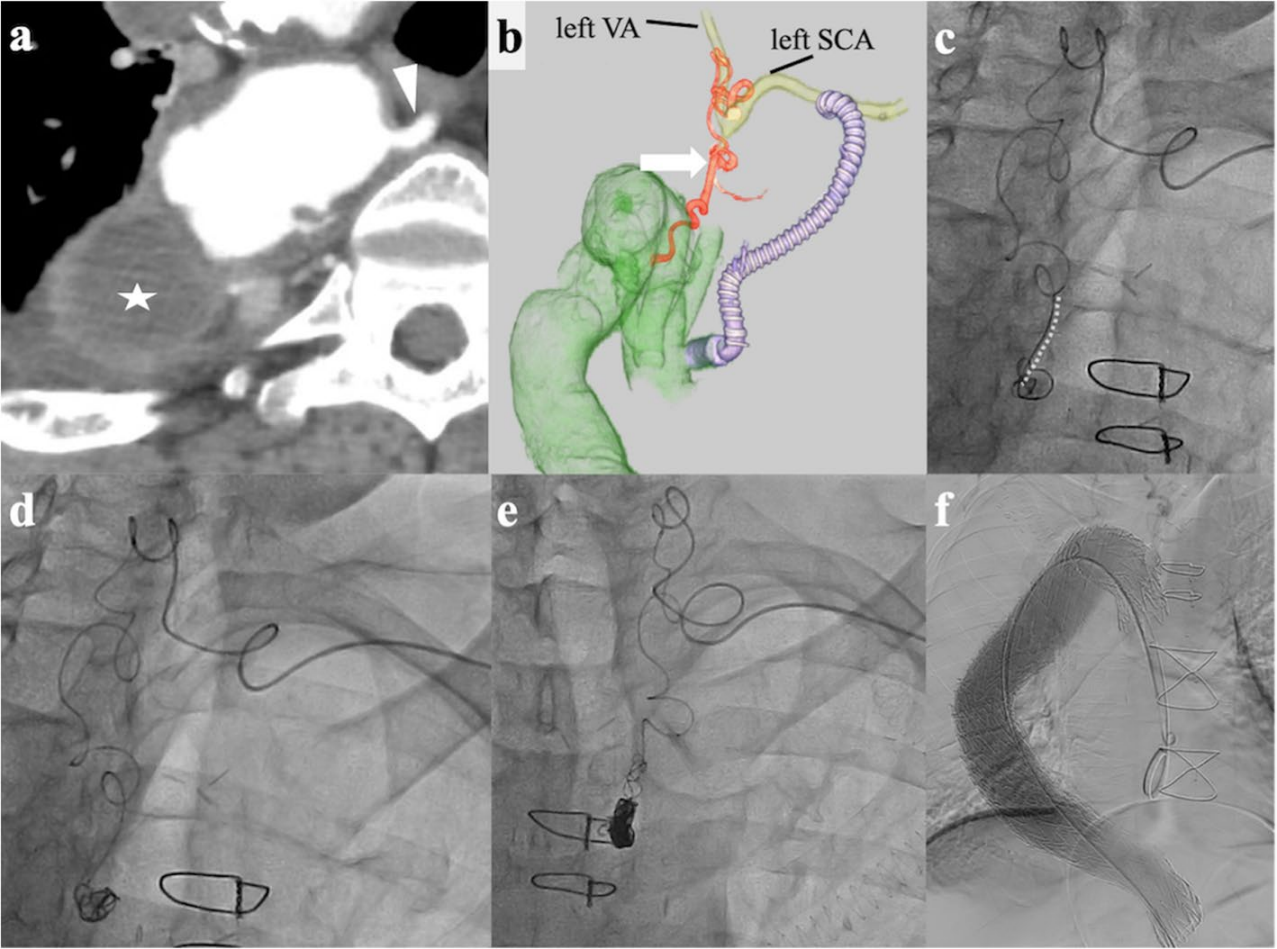
A 69-year-old male presented with an extensive thoracic aortic aneurysm, a right aortic arch, and an isolated left subclavian artery (SCA). Initially, we performed an elephant trunk ascending aorta replacement and reconstructed the cervical branches. Subsequently, we planned for thoracic endovascular aortic repair (TEVAR) to address the aortic arch and descending aortic aneurysm. **a** contrast-enhanced computed tomography revealed a dilated third intercostal artery measuring 4 mm (arrowhead) and a coarse mural thrombus within the aneurysm (star). Embolization of the artery was planned to prevent a type 2 endoleak; **b** a three-dimensional volume-rendering image demonstrated that the highly tortuous third intercostal artery (arrow) anastomosed with the left costocervical trunk, which arises from the left SCA, and that the left vertebral artery (VA) branched off closely to the left costocervical trunk. Considering the risk of peripheral embolization of the VA due to a substantial mural thrombus, we punctured the left brachial artery and accessed this artery from the left SCA; **c** a 1.6-Fr microcatheter (Carnelian Marvel S; Tokai Medical Products, Aichi, Japan) was advanced near the origin of the third intercostal artery, and we attempted to place a 0.014-inch coil (Target XL soft coil; Stryker, Fremont, CA, USA) measuring 5 mm in diameter and 10 cm in length. However, the pathway’s tortuosity was so severe that we could not implant the entire coil, and half of it remained within the catheter (dotted line); **d** therefore, the coil was intentionally detached within the catheter and flushed with saline solution for placement; **e** seven additional 0.014-inch coils (six target XL soft coils and one Azur soft 3D coil; Terumo, Tokyo, Japan) were tightly placed using the intentional early detachment technique; **f** final angiogram after TEVAR shows successful aortic repair without any endoleaks

In the tortuous vascular anatomy scenario, the targeted vessels included the third intercostal artery through the costocervical trunk (*n* = 1) (patient number 2, Fig. 4), the posterior inferior pancreaticoduodenal artery (*n* = 1) (patient number 4), and the fourth lumbar artery through the iliolumbar artery (*n* = 2) (patient number 10 and 11). The tortuosity of the vessels made it challenging to push the delivery wire of the detachable coils through the microcatheters. Liquid embolic agents were deemed inappropriate due to the anastomoses observed in critical vessels such as the vertebral artery (patient number 2), the pancreaticoduodenal arcades (patient number 4), and the spinal branches of lumbar arteries (patient number 10 and 11). The intentional early detachment technique allowed for precise coil placement in these cases. Figure 4 shows a representative case.

**Fig. 5:**
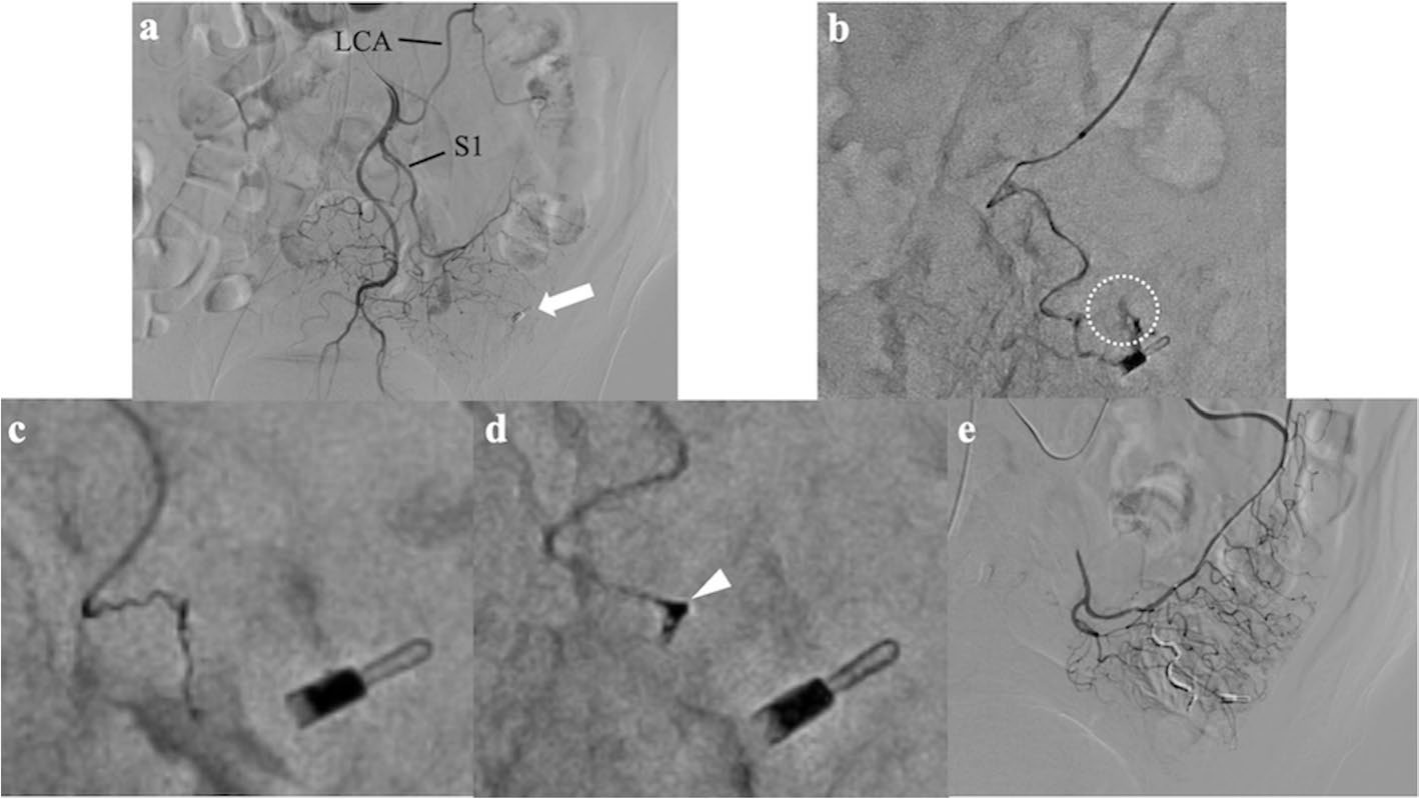
An 86-year-old male presented with ascending colonic hemorrhage due to colonic diverticulosis. Transcatheter arterial embolization performed after hemostatic clipping through colonoscopy was unsuccessful. **a** inferior mesenteric angiography revealed small and significantly tortuous vasa recta of the sigmoid artery (arrow) without contrast extravasation near clipping (arrow); **b** superselective angiography through the vasa recta of the first sigmoid artery (S1) with a 1.6-Fr microcatheter (Carnelian Marvel S; Tokai Medical Products, Aichi, Japan) showed contrast extravasation at the distal end of near clipping (circle); **c** an attempt was made to place a 0.010-inch coil (the COMPLEX SilkySoft iED coil; KANEKA Medix, Osaka, Japan) measuring 1 mm in diameter and 3 cm in length, but the catheter kicked back, and it could not be placed tightly. Consequently, the coil was retrieved without detachment; **d** the same coil was placed using the intentional early detachment technique and was, therefore, placed tightly (arrowhead); **e** five additional 0.011-inch coils (Avenir; Wallaby Medical, Shanghai, China) were placed using the same technique, and the final angiogram did not reveal any contrast extravasation

In the inadequate system backup scenario, the targeted vessels were the dorsal pancreatic artery (*n* = 1) (patient number 5), the iliolumbar artery (*n* = 1) (patient number 8), and the vasa recta of the sigmoid artery (*n* = 1) (patient number 9). The microcatheters exhibited kickback during coil placement, preventing the tight packing of the coils and risking catheter dislodgment. Liquid embolic agents were deemed inappropriate in these cases due to the following reasons: in patient number 9, the vasa recta was very thin, and the slow contrast injection easily caused reflux, suggesting a high risk of non-target embolization; in patient number 5, the peripheral vessels anastomosed with the pancreaticoduodenal arcade; and in patient number 8, using liquid embolic agents could result in an unnecessarily extensive embolization range of the entire iliolumbar artery. However, the intentional early detachment technique ensured a secure coil placement without catheter kickback. Figure 5 demonstrates a representative case.

**Fig. 6:**
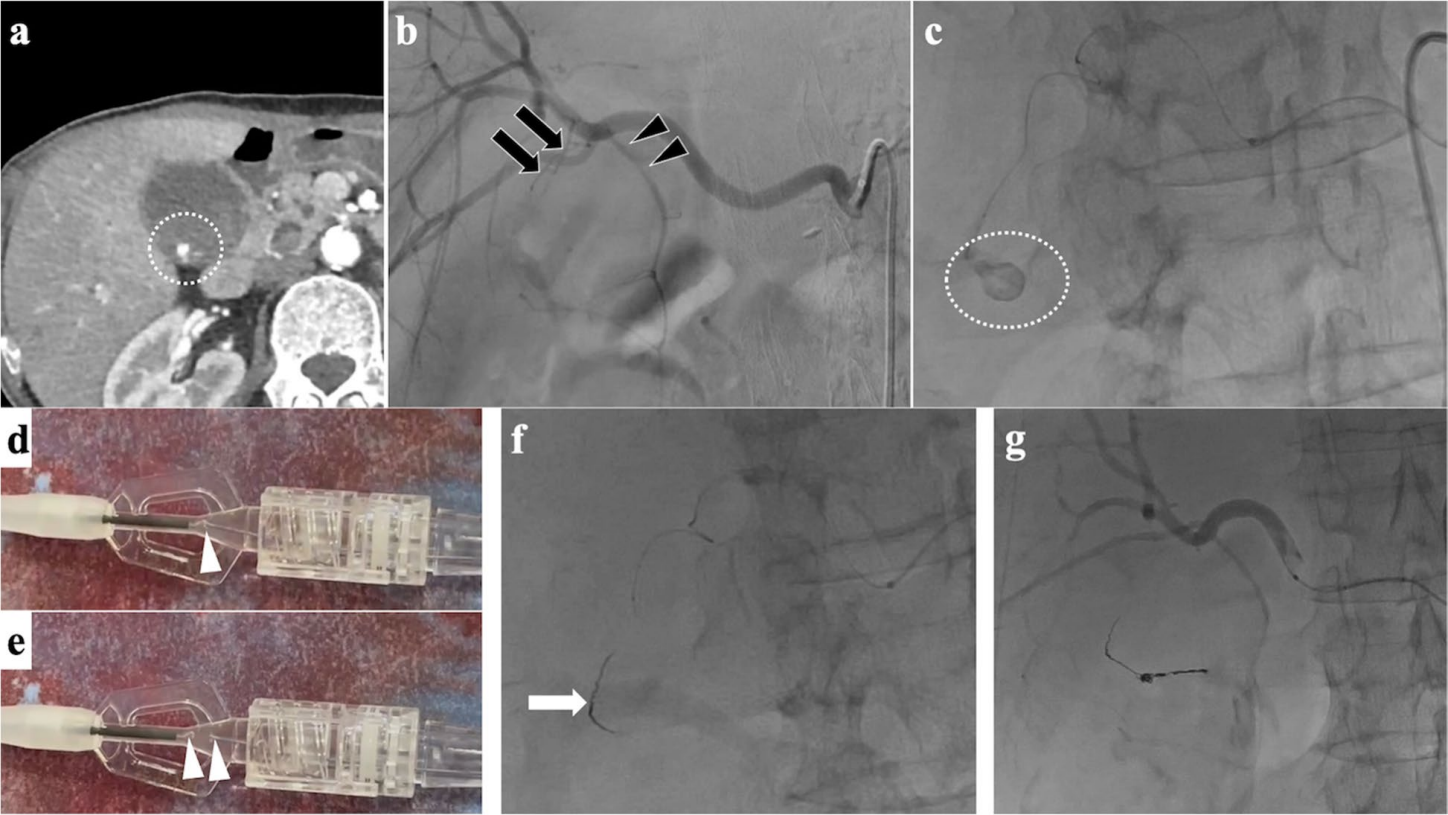
A 69-year-old male presented with hematemesis. **a** contrast-enhanced computed tomography demonstrated pseudoaneurysm in the gallbladder (circle); **b** angiography from the replaced right hepatic artery revealed two cystic arteries (black arrow and arrowhead); **c** the selection of the right-sided cystic artery (black arrow) was difficult, and a 1.3-Fr microcatheter (DeFrictor Nano; Medico's Hirata, Osaka, Japan) was used. Superselective angiography through the cystic artery showed a pseudoaneurysm (circle); **d** and **e** advancing just the coil section of the 0.010-inch Target Nano coil (Stryker, Fremont, CA, USA), which measures 1 mm in diameter and 3 cm in length, into the microcatheter and ensuring that the detachment point is slightly within the catheter hub (white arrowhead), allows for successful detachment; **f** two 0.010-inch coils were placed (white arrow) using the intentional early detachment technique. The left-sided cystic artery was easily selected with a 1.6-Fr catheter (Carnelian MARVEL S; Tokai Medical Products, Aichi, Japan) and embolized with three detachable coils using the standard method; **g** angiography after embolization revealed the complete embolization of the pseudoaneurysm

In the 1.3-Fr microcatheter scenario, the targeted vessels were small and tortuous, including the anterior inferior pancreaticoduodenal artery (*n* = 1) (patient number 1), the cystic artery (*n* = 1) (patient number 3), the second lumbar artery through the first lumbar artery (*n* = 1) (patient number 6), and the posterior inferior pancreaticoduodenal artery (*n* = 1) (patient number 7). Liquid embolic agents were deemed inappropriate due to the anastomoses in critical vessels, such as the pancreaticoduodenal arcades (patient number 1 and 7) and the spinal branches of the lumbar artery (patient number 6). In the case of the cystic artery (patient number 3), we decided that coil embolization was more appropriate, considering that using liquid embolic agents might lead to an extensive embolization range and a higher risk of cholecystitis. Successful embolization was achieved using 10-coils (0.0095–0.011 inches in primary diameter) in 1.3-Fr microcatheters. The 10-coils delivery wire is thicker than the lumen of the 1.3-Fr catheter, but the coil portion can be fully inserted into the catheter. Therefore, we advanced the 10-coil through the 1.3-Fr microcatheter to a point where the delivery wire could enter without resistance. We detached the coil and then flushed it with saline. This technique allowed us to embolize with 10-coils, even with a 1.3-Fr microcatheter. Figure 6 illustrates a representative case.

The technical and clinical success rates were 100% in all scenarios. No coil sticking in the microcatheter, malposition in the target vessel, or migration into non-target vessels occurred. Similarly, no major complications were observed. All patients achieved satisfactory clinical outcomes, symptom resolution, and successful management of their underlying conditions.

## Discussion

When faced with tortuous and meandering vascular pathways, the stiffness of the delivery wires can sometimes impede the complete deployment of detachable coils within the vessel [10]. Using coils from different manufacturers, even those with distinct characteristics can be challenging in such scenarios. Improving the backup support system is plausible [11]; however, this method has limitations when navigating thick-diameter catheters through highly tortuous pathways [12]. The effectiveness of triaxial systems for navigating through bent pathways has also been reported [12, 13]; however, we used triaxial systems in all our cases and could not place the coils using standard methods. Embolization with pushable coils using a saline flushing technique is effective [14–16]; however, it may not be suitable in cases lacking appropriately sized coils that are feasible for large vessel diameters. Typically used pushable coils, such as the Nester, Tornado, and Hilal coils (Cook Medical, Bloomington, IN, USA), are mainly designed for 0.018-inch inner lumen catheters and cannot be deployed through the 1.6-Fr or 1.3-Fr microcatheters used in our study, which have inner lumens of 0.014 and 0.011 inches, respectively. In Japan, 0.014-inch pushable coils (C-STOPPER coil; Piolax, Kanagawa, Japan) with a diameter of 2.0–2.8 mm are available for use with 1.6-Fr microcatheters. The effectiveness of super-selective coil embolization using these coils through the 1.6-Fr microcatheter of the triaxial system has been reported [13]. However, the C-STOPPER coil has a limited diameter range, and achieving tight placement may be impossible when used in vessels significantly < 2.0 mm, resulting in linear placement and insufficient embolization. Moreover, the migration risk is high in vessels with diameters > 2.8 mm, limiting the use of pushable coils. In such situations, intentionally detaching a larger-diameter detachable coil within the microcatheter and subsequently flushing it with saline allows for a secure placement without the migration risk (Figs. 4c and d).

In cases where the backup support is inadequate, the kickback effect of the microcatheter during placement can prevent tight embolization or lead to catheter deviation into the mother vessel, even if the entire length of the coil can be delivered from the catheter. Under such circumstances, the intentional early detachment technique with the detachable coils can reliably achieve a tight placement. In one of our patients (Fig. 5), we initially attempted to place an iED coil measuring 1 mm in diameter and 3 cm in length in the vasa recta of the first sigmoid artery. However, we only achieved loose placement due to difficulties in catheter control and kickback (Fig. 6c). We retrieved the coil, intentionally detached it, and flushed it with saline, resulting in tight embolization (Fig. 6d).

In scenarios involving highly tortuous vessel pathways requiring ultra-thin microcatheters with a distal tip size of ≤ 1.5-Fr, these microcatheters are designed for use with liquid embolic materials and are essentially incompatible with coils. Previous reports have indicated that detachable coils can be used with a 1.5-Fr Marathon microcatheter (Medtronic, Minneapolis, MN, USA) [10, 17, 18]. The inner lumen at the tip is as small as 0.013 inches, limiting the types of coils that can be used. The inner lumen of microcatheters with a distal tip size of 1.3-Fr, such as the DeFrictor Nano (Medico's Hirata, Osaka, Japan) and Marvel S 1.3 (Tokai Medical Products, Aichi, Japan), is even narrower (0.011 inches). In our experience, standard methods cannot be used to deploy coils through narrow-lumen microcatheters. This limitation is because the diameter of the coil’s delivery is larger than that of the inner lumen of these microcatheters. However, as described in the Results section, we successfully achieved embolization with 10-coils in 1.3-Fr microcatheters using the intentional early detachment technique. Notably, some electrically detachable coils may not be detachable within the catheter as the detachment point must be energized for detachment and requires surrounding electrolytes, including blood or saline solution. Consequently, detachment within the catheter may not always be feasible using coils such as the iED and Target coils. The Target coils can sometimes be detached within a 1.6-Fr catheter after multiple detachment attempts; however, they often cannot be detached within a 1.3-Fr catheter. This is due to the narrow lumen leading to insufficient electrolytes around the detachment point.

To date, there are no established guidelines on selecting embolic agents for embolization procedures. The selection of the embolic materials depends on the encountered anatomy, tortuosity, and caliber of the arteries, the technique chosen, and the operator's confidence with the available materials [19]. Each embolic agent is characterized by its strengths and weaknesses and can be used alone or combined with others to enhance its embolic power [19]. Even in situations where standard coil embolization is challenging, the use of other embolic agents, such as microplugs, gelatin sponges, or liquid embolic agents (n-butyl-2- cyanoacrylate and ethylene vinyl alcohol copolymer), should be considered before employing our technique. The recommended minimum microcatheter inner lumen for microvascular plugs is 0.021 inches, making their use difficult with thin microcatheters. However, injecting liquid embolic agents was possible in all our cases. We considered using liquid embolic agents inappropriate due to the presence of important peripheral vascular anastomoses, the risk of easy reflux in thin vessels leading to a high risk of non-target embolization, or the potential for unnecessarily extensive embolization. However, this decision ultimately depends on the operator's judgment on a case-by-case basis, and clear criteria will need to be established in the future. Therefore, it is crucial to carefully consider the potential risks associated with our technique. Catheter dislocation during coil flushing and the difficulty of retrieving detached coils in complex anatomies are critical concerns. Careful consideration and operator experience are essential when employing this technique, and a contingency plan should be in place in case of complications.

This study has some limitations. It was a retrospective study conducted at a single facility and included a limited number of patients. The intentional early detachment technique with detachable coils is off-label, and the safety and efficacy of this technique have not been thoroughly evaluated. We did not encounter any complications in our limited case series; however, the potential risks associated with this off-label use should be carefully considered. Consequently, if 0.010-inch or 0.014-inch pushable coils with larger diameters become commercially available, they might be alternatives to the intentional early detachment technique with detachable coils. The detachable coils in the described technique are employed similarly to how pushable coils would be used, meaning that the coil's position cannot be adjusted through repeated retrieval and repositioning during deployment. Additionally, detachable coils are more expensive than pushable coils, and using this technique can significantly increase the cost of the procedure. However, this study underscores the potential utility of alternative techniques in situations where conventional approaches are limited by vascular anatomy or equipment availability.

## Conclusion

This study demonstrates the effectiveness of the intentional early detachment technique with detachable coils in complex vascular embolization scenarios. The method has been successfully applied in challenging cases, enabling coil placement in difficult vascular embolization situations. Its applicability is limited to specific scenarios; however, this technique offers interventionalists a valuable option when faced with anatomical or equipment constraints.

## Data Availability

Data sharing does not apply to this article, as no datasets were generated or analyzed in the current study.
